# Role of Reactive Oxygen Species and Nitric Oxide in Mediating Chemotherapeutic Drug Induced Bystander Response in Human Cancer Cells Exposed In-Vitro

**DOI:** 10.4021/wjon474w

**Published:** 2012-04-23

**Authors:** Mani Chinnadurai, Bhavna S Rao, Ramasamy Deepika, Solomon F.D. Paul, Perumal Venkatachalam

**Affiliations:** a Department of Human Genetics, College of Biomedical Science Technology and Research, Sri Ramachandra University, Porur, Chennai, India

**Keywords:** Bystander effect, Bleomycin, Neocarzinostatin, Reactive oxygen species, Nitric oxide, Micronucleus assay

## Abstract

**Background:**

The intention of cancer chemotherapy is to control the growth of cancer cells using chemical agents. However, the occurrence of second malignancies has raised concerns, leading to re-evaluation of the current strategy in use for chemotherapeutic agents. Although the mechanisms involved in second malignancy remain ambiguous, therapeutic-agent-induced non-DNA targeted effects like bystander response and genomic instability cannot be eliminated completely. Hence, Bleomycin (BLM) and Neocarzinostatin (NCS), chemotherapeutic drugs with a mode of action similar to ionizing radiation, were used to study the mechanism of bystander response in human cancer cells (A549, CCRF-CEM and HL-60) by employing co-culture methodology.

**Methods:**

Bystander effect was quantified using micronucleus (MN) assay and in-situ immunofluorescence(γH2AX assay).The role of reactive oxygen species (ROS) and nitric oxide (NO) in mediating the bystander response was explored by pre-treating bystander cells with dimethylsulphoxide (DMSO) and C-PTIO respectively.

**Results:**

Bystander response was observed only in CCRF-CEM and A549 cells (P < 0.001). A significant decrease in this response was observed with ROS scavenger, DMSO.

**Conclusion:**

This significant attenuation in the bystander response on treatment with DMSO, suggests that ROS has a more significant role in mediating the bystander response.Since the possibility of the ROS and NO in mediating these bystander effect was confirmed, mechanistic control of these signaling molecules could either reduce radiation damage and potential carcinogenicity of normal tissues (by reducing bystander signaling) or maximize cell sterilization during chemotherapy (by amplifying bystander responses in tumors).

## Introduction

There is now an extensive body of evidence for the spatial and temporal transmission of adverse effects from irradiated cells to un-irradiated ‘‘bystander’’ cells, and the phenomenon is termed as bystander response. Bystander effects were found to increase genomic instability and reduce cell survival in un-irradiated cells. This calls for re-evaluation of therapeutic strategies in order to obtain better response and reduce the risk of therapy associated second tumors.

Studies have shown that radiation-induced bystander effects are mediated either through transmission of certain biomolecules and/or molecular signals either by intercellular communication through gap junctions [1] or by release of soluble factors into the culture medium [2]. Even though molecules like reactive oxygen species (ROS) [3], interleukin 8 [4], transforming growth factor beta (TGFβ) [5], tumor necrosis factor α (TNF α) [6], tumor necrosis factors related apoptosis inducing ligand (TRAIL), 5-hydroxytryptamine, L-DOPA, glycine, or nicotine [7, 8] and nitric oxide (NO)[9] have been shown to mediate radiation-induced bystander effects; the mechanism is yet to be identified. Similarly, signaling pathways of cyclooxygenase-2 (COX-2) [10] and Nuclear Factor KappaB (NFkB) [11] have been shown to be involved in mediating bystander responses.

Emerging literature has shown that cancer chemotherapeutic drugs like mitomycin [12], actinomycin [13] and bleomycin [14] can also induce bystander response and genomic instability [15] in unexposed neighboring cells similar to ionizing radiations. In line with these results, mitomycin C and phleomycin induce normal human B-lymphoblastoid cells to produce medium soluble factors that elicit bystander effects in unexposed cells [12]. Molecular players involved in mediating radiation and chemical induced bystander response are diverse and may depend on the cell type or its physiological state and genotoxic agent used. Similar to radiation-induced bystander response, the mediator of the chemotherapeutic bystander effect has not been identified yet but is of potential therapeutic interest. Although ROS and NO were shown to be the mediators of chemotherapeutic drug, bleomycin(BLM) induced bystander response in normal cells (human mesenchymal stem cells and peripheral blood lymphocytes)[14], the real players in the case of cancer cells are yet to be identified. Hence it was of interest to investigate the role of ROS and NO in mediating the bystander response induced by chemotherapeutic drugs, BLM and neocarzinostatin (NCS) in human cancer cells namely A549 (lung adenocarcinoma), CCRF-CEM (T-lymphoblast like) and HL-60 (Human leukemia) cells.

## Materials and Methods

### Maintenance of cell lines

The cell lines used, namely, A549, CCRF-CEM and HL-60, obtained from NCCS, Pune, were maintained and grown in a humidified incubator at 37°C with 5% CO_2_. While the A549 cells were grown as a monolayer in plastic tissue culture flasks (p24 to p38) in Dulbecco’s Modified Eagles Medium (DMEM) (GIBCO, Grand Island, New York, USA), HL-60 (p6 to p12) and CCRF-CEM (p8 to p12) cells were grown as suspension cultures in Minimum Essential Medium (MEM) (GIBCO, Grand Island, New York, USA). The media was supplemented with 10% fetal bovine serum (FBS) (GIBCO, Grand Island, New York, USA) and antibiotics (Penicillin 50 IU/mL, Streptomycin 35 mg/mL and Gentamycin 2.5 mg/mL) (GIBCO, Grand Island, New York, USA).

### Co-culture of BLM and NCS exposed cells with unexposed cells

Approximately 1 x 10^5^ cells were seeded onto commercially available transwell culture inserts (Thincert™, Greiner Bio one, Germany) and an equal number of cells were seeded into six well plates. This was incubated at 37°C for 24 hours. After 24 hours, the cells in the thincert were exposed to the drug BLM (Dabur, Solan, India) at concentrations of 40 and 80 mg/mL for 3 hours and NCS (Sigma, Rehovot, Central District, Israel) at concentrations of 0.5 and 2 mg/mL for 1 hour. A set of controls was used. Following exposure, the drug treated cells were washed thrice with their respective media. The cells in the thincerts (directly exposed) and in the six well plates (unexposed or bystander) were co-cultured using the methodology described by Geraschenko and Howell [16].

### Analysis of DNA damage using micronucleus (MN) assay

Following co-culture,1 x 10^5^ directly exposed and bystander cells were seeded onto p60 plates and cultured in their respective media with 10% FBS and cytochalasin-B (3 mg/mL) (Sigma, Bellefonte, USA) to arrest cells at the cytokinesis stage at 37°C in a 5% CO_2_ incubator. The adherent cells were harvested after 72 hours and the suspension cultures after 48 hours. For suspension cultures, the cells were subject to a brief treatment with hypotonic solution (0.45% KCl) and fixed with methanol and acetic acid in the ratio 3:1. The cells were dropped on cold glass slides, stained with 10% Giemsa solution (pH 6.8, GIBCO, Burlington, USA) and analysed under a light microscope. The adherent cells were washed with phosphate buffer saline (PBS), fixed with ice-cold methanol and air dried after 72 hours of incubation. The cells were stained with diamino-phenyle-indole (DAPI) (VysisInc, Downers Grove, USA) and visualized using a fluorescence microscope.

Binucleated cells (cells with two daughter nuclei surrounded by cytoplasm) were scored for the presence of MN according to the scoring criteria given by Fenech et al [17]. Based on the recorded data, the frequency of MN (MNi) was calculated using the formula MNi = a/b and the error was calculated by MNi = √a/b where ‘a’ is the total number of micronuclei and ‘b’ is the total number (1000) of binucleate cells scored.

### Quantification of bystander effect by in-situimmuno-fluorescence assay

Following co-culture, about 1 x 10^6^ directly exposed and bystander cells were taken in separate tubes for flow cytometric evaluation of γH2AX and COX-2. The cells were fixed with 2% paraformaldehyde and ice-cold methanol, and made permeable by treatment with 1% Triton X-100. The cells were blocked in 1% Bovine Serum Albumin (BSA) for 1 hour, washed and incubated at 4°C overnight with primary antibody for γH2AX and COX-2 (Abcam, Ab 1431-100 and Ab 90345, respectively). After overnight incubation, the cells were washed to remove excess primary antibody and incubated with the respective FITC labeled secondary antibody (Abcam, Ab 6717 and Ab 6785 respectively) for 1hr at 37°C in the dark. Flow cytometry measurement was done using a FACS Calibur (Becton Dickinson Systems) and 10,000 events were analyzed using Cell Quest software. The relative FITC fluorescence intensities were calculated by taking the ratio of the histograms representing exposed cells and control cells [18].

### Effect of Dimethyl sulphoxide (DMSO) and C-PTIO on bystander cells

As DMSO and C-PTIO are known scavengers of ROS and NO, respectively, the role of ROS and NO in mediating the drug induced bystander effect was studied. Prior to co-culture of directly exposed and unexposed cells, the unexposed cells were pretreated with 10mM DMSO (Sigma, USA) or 20 mM C-PTIO (Sigma, USA) respectively for an hour and the DNA damage was measured using MN assay.

### Statistical analysis

The differences in the MN frequencies and relative fluorescence intensity ratios within and between the treatment groups for each cell type were compared. As all experiments were repeated thrice (n = 3), the average of the assimilated data was compared by the paired t-test and one-way analysis of variance (ANOVA) using the INSTAT programme.

## Results

### Analysis of DNA damage in BLM exposed and bystander cells using MN and γH2AX assay

**Figure 1:**
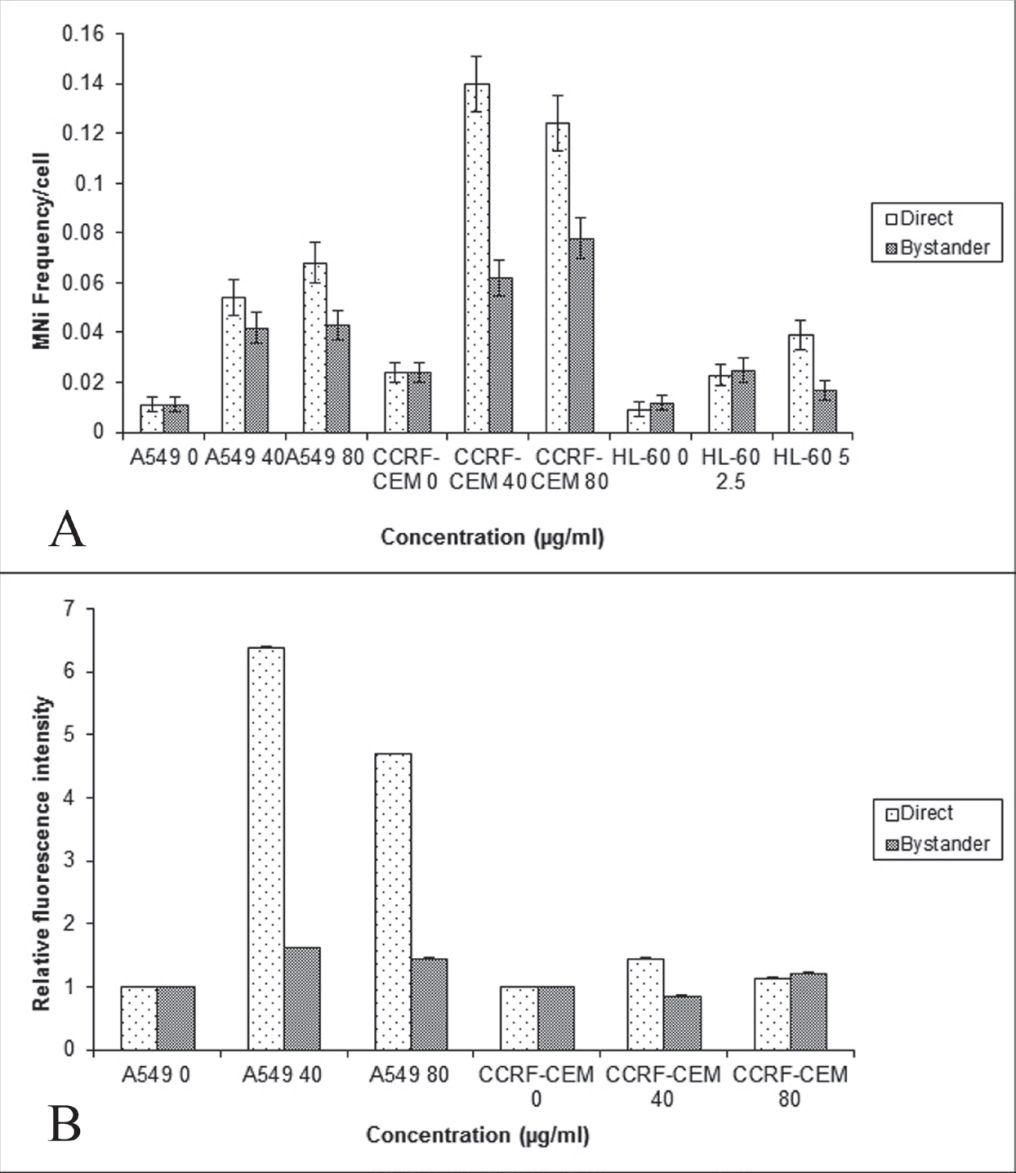
Comparison of the MN frequency in the A549, CCRF-CEM and HL-60 cells (A) and γH2AX relative fluorescent intensity (B) obtained using flow cytometry in the A549 and CCRF-CEM cells that are exposed to BLM and then co-cultured with their respective bystander cells for 24hrs. Each bar represents the mean ± SE of the frequency of micronuclei and relative fluorescence intensity induced for three independent experiments (n = 3).

Baseline MN frequencies obtained in the direct control cells of A549, CCRF-CEM and HL-60 cells were 0.011 ± 0.003, 0.024 ± 0.004, and 0.009 ± 0.003 respectively (standard errors of the mean (SEM) for n = 3). Of the three cell lines used, the CCRF-CEM lymphoblast like cells had a higher base line MN frequency. No variation was observed in the baseline MN frequencies of direct and bystander control cells**.** The various cell types (A549, CCRF-CEM and HL-60 cells) used in the study were exposed to a concentration of 40 and 80 mg/mL BLM for three hours and the DNA damage was quantified using the MN assay and γH2AX assay. The obtained results are provided in Figure 1. When HL-60 cells were exposed to 40 and 80 mg/mL of BLM, the cells underwent apoptosis (data not shown). Hence the concentration of BLM was reduced to 2.5 and 5 mg/ml and then used for co-culture experiments. The result showed that BLM treatment induced a significant increase in the MN frequency (P < 0.001) in A549, CCRF-CEM and HL-60 cells (Fig.1A). However, only bystander A549 and CCRF-CEM cells showed a significantly (P < 0.001) higher MN frequency when compared to their respective controls, but the induced MN frequency did not show any dose dependency. Thus, the observed higher frequency of MN in the bystander cells suggests that the BLM induced bystander effect is similar to ionizing radiation and concentration independent. HL-60 cells showed an increased MN frequency in the cells directly exposed to BLM. But in the bystander cells, only the bystander 40 µg/mL showed increased MN frequency when compared to its control.

As the co-cultured bystander cells of A549 and CCRF-CEM cells alone showed enhanced DNA damage as measured by the MN assay, the bystander response was further examined in these cell lines by quantifying another marker of DNA damage, the γH2AX foci. The γH2AX assay showed an increase in the fluorescence intensity in both directly exposed (P < 0.001) and bystander cells of A549, and in the bystander cells of 80 µg/mL CCRF-CEM (Fig. 1B); further confirming that BLM exposure could induce bystander response in unexposed A549 and CCRF-CEM cells.

### Analysis of DNA damage in NCS exposed and bystander cells using MN and γH2AX assay

**Figure 2:**
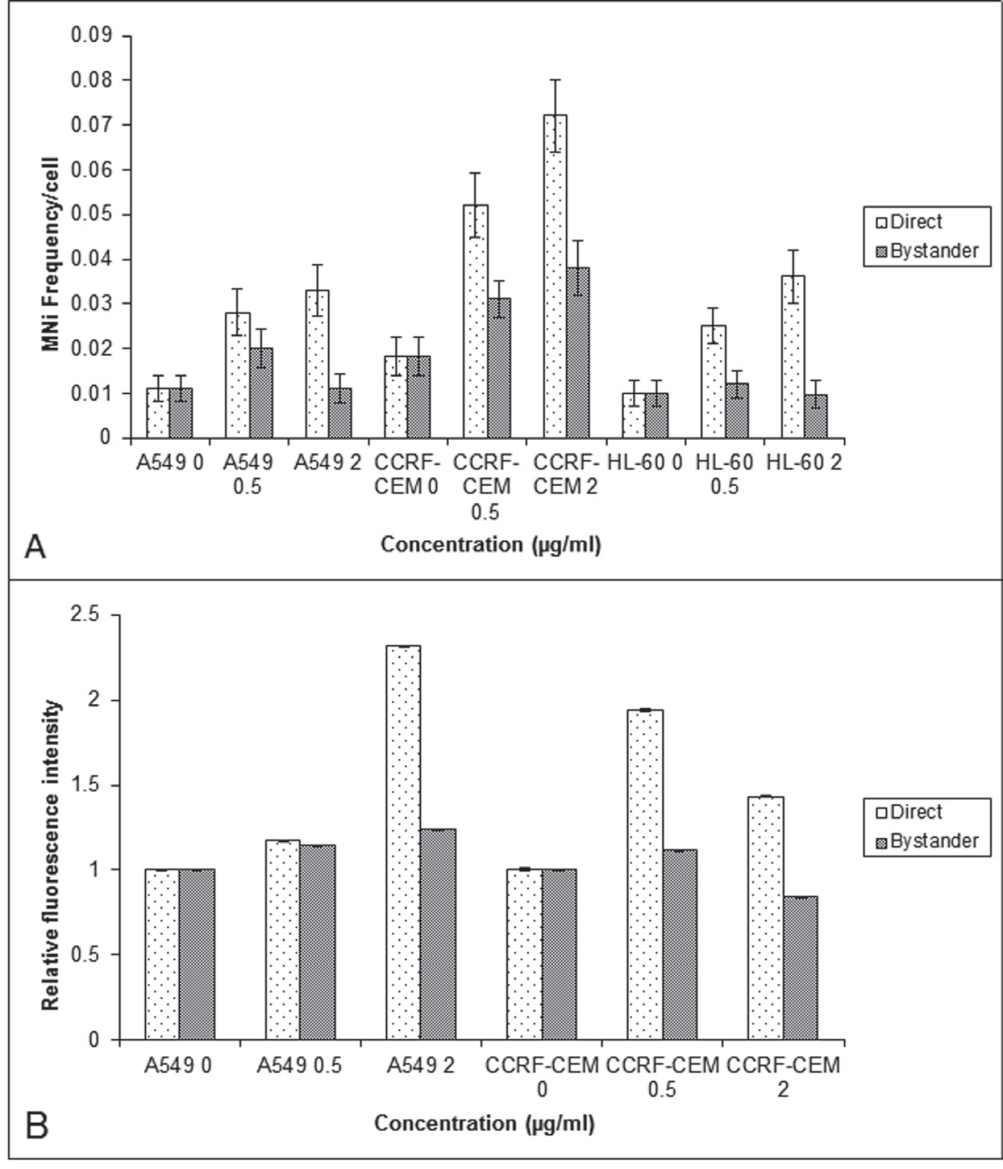
Comparison of the MN frequency in the A549, CCRF-CEM and HL-60 cells (A) and γH2AX relative fluorescent intensity (B) obtained using flow cytometry in the A549 and CCRF-CEM cells that are exposed to NCS and then co-cultured with their respective bystander cells for 24 hrs. Each bar represents the mean ± SE of the frequency of micronuclei and relative fluorescence intensity induced for three independent experiments (n = 3).

The study of BLM induced bystander effect, observed in A549, CCRF-CEM and HL-60 was extended further by using another radiomimetic drug, NCS. NCS induced DNA damage was measured using the MN (Fig. 2A) and γH2AX assays (Fig. 2B). In A549 and CCRF-CEM, the MN frequency of directly exposed and bystander cells were significantly higher (P < 0.05) than their respective controls. However in HL-60, the MN frequency showed a significant increase only in directly exposed cells (P < 0.05) indicating the absence of NCS bystander response in HL-60 leukemic cells (Fig. 2A).

γH2AX evaluation showed an increase in the fluorescence intensity in directly exposed as well as the bystander cells of A549, and in the bystander cells of 40 µg/mL CCRF-CEM(P < 0.01) (Fig. 2B).

### Effect of ROS and NO scavengers in mediating the bystander effect

**Figure 3:**
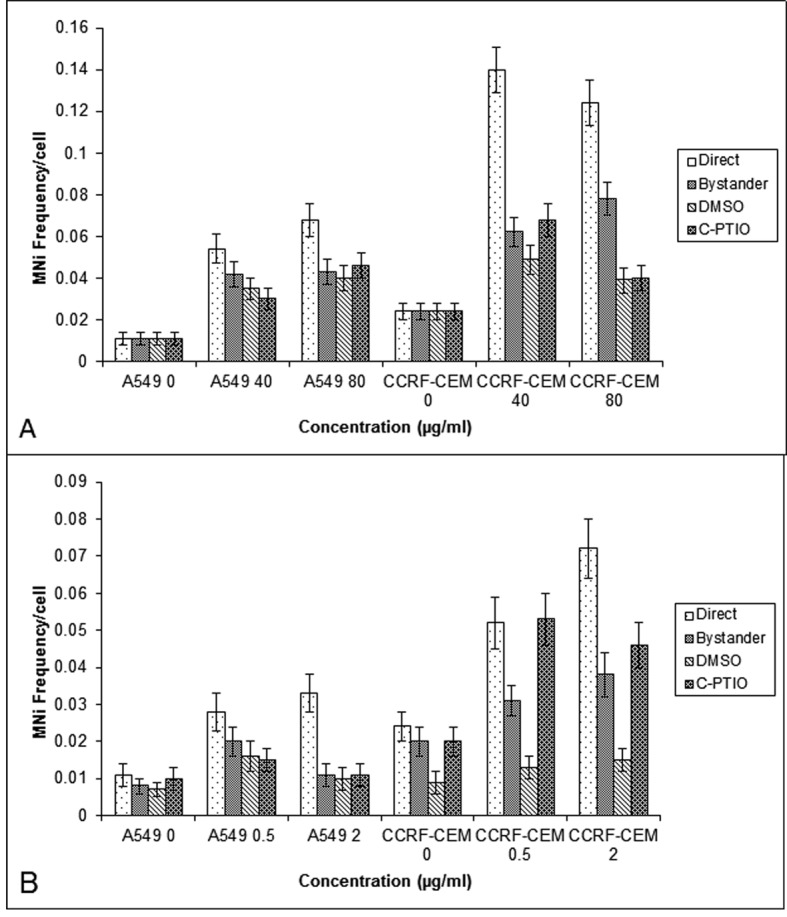
MN frequency obtained in the A549 and CCRF-CEM cells that are exposed to BLM (A) or NCS (B) and then co-cultured with their respective bystander cells for 24 hrs with and without DMSO or C-PTIO. Each bar represents the mean ± SE of the frequency of micronuclei induced for three independent experiments (n = 3).

Since it has been suggested that ROS and NO are involved in mediating radiation induced bystander response [19], its role was studied in A549 and CCRF-CEM cells. The cells were pretreated with DMSO (10 mM) and C-PTIO (20 mM) and co-cultured with cells which had been directly exposed to BLM and NCS. Pre-treatment of control bystander cells did not alter the baseline MN frequency. Pretreated bystander A549 cultures displayed an attenuation of bystander response at low concentration of BLM (40 mg/mL) and NCS (0.5 mg/mL); however, this reduction was statistically insignificant (P > 0.05). Alternatively in CCRF-CEM cells, the pre-treatment of bystander cells with DMSO, reduced the MN frequency significantly (P < 0.001) in bystander cells co-cultured with BLM exposed cells (40 and 80 mg/mL) as well as NCS exposed cultures (0.5 mg/mL and 2 mg/mL). Whereas, the pre-treatment with C-PTIO did not show any decrease in MN frequency (P > 0.05) in bystander cells, except 80 µg/mL BLM bystander cells (Fig. 3A and B).

### Expression of COX-2 in cells exposed to BLM, NCS and their respective bystander cells

**Figure 4:**
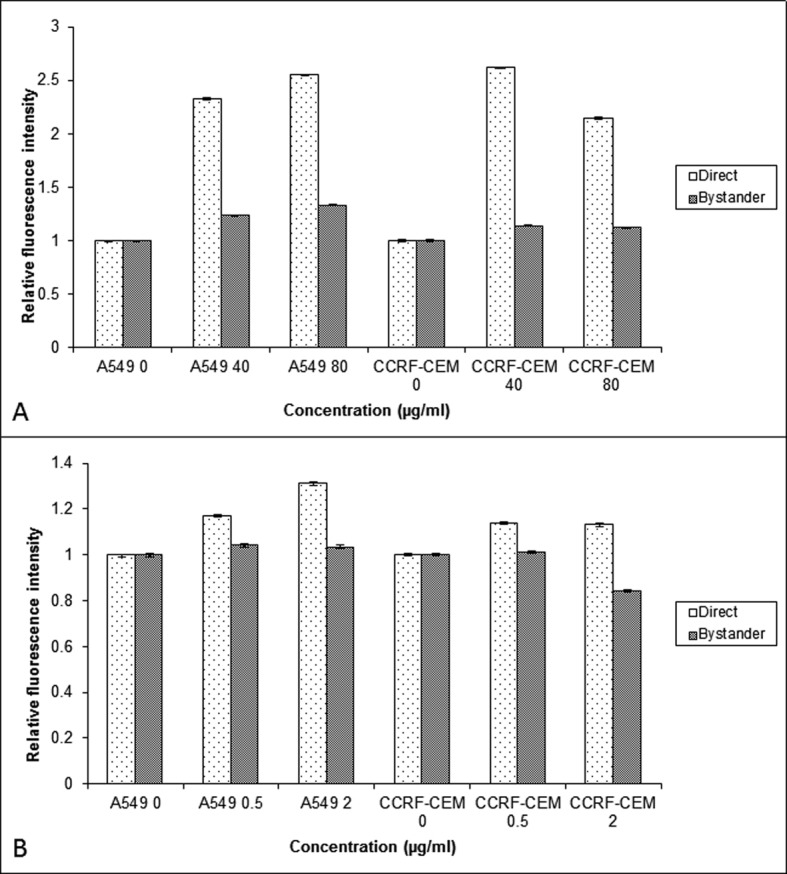
Comparison of the COX-2 relative fluorescent intensity obtained using flow cytometry in the A549 and CCRF-CEM cells that are exposed to BLM (A) and NCS (B) and then co-cultured with their respective bystander cells for 24 hrs. Each bar represents the mean ± SE of the relative fluorescence intensity induced for three independent experiments (n = 3).

To find out molecular mechanisms mediating the bystander effect, the COX-2 level was measured in CCRF-CEM and A549 cells exposed to BLM or NCS. The COX-2 expression was higher in the BLM directly exposed cells when compared to that of NCS. The bystander cells (A549 and CCRF-CEM) co-cultured with BLM exposed cells showed increased expression of COX-2 when compared to their control (P < 0.001) (Fig. 4A). In contrast, no increased expression of COX-2 was observed in the bystander cells exposed to NCS treated cells (P > 0.05) (Fig. 4B).

## Discussion

Chemotherapy and radiotherapy are commonly used in the treatment of cancer with the use always based on the rationale of risk against benefit. Though, generally the benefits outweigh the risks in cancer therapy, there is a raised concern on the increased incidence of therapy induced second malignancy. Second malignancies, after radiotherapy [20] or combined radio and chemotherapy [21] are seen more frequently in younger individuals compared to adults, which has now become the leading cause of death among 15 year survivors of lymphoma. Alkylating chemotherapy has been shown to cause a large relative risk of leukemia within a few years after treatment. Similarly, risks from solid tumors increase more gradually, but in the long term, these cancers constitute the great majority of second malignancies, and the extent of these risks and their etiology are incompletely understood. Hence both radiotherapy and chemotherapy are themselves carcinogenic and second malignancy after treatment is important to characterize and prevent.

We speculate that an increased risk of second cancer may in part reflect the induction of genome destabilizing mutations in bystander cells due to non-targeted effects of therapeutic agents, as it has been shown that radiation and chemotherapeutic agents can induce bystander effects and genomic instability [22]. Involvement of ROS appears to be common to both, radiation induced genomic instability and bystander effect [23]. Hence, we investigated the role of ROS and NO in lymphoblastoid and lung adenocarcinoma cells of human origin, in mediating BLM and NCS induced bystander effects. The CCRF-CEM cells were selected as they are T-lymphoblast like cells constantly in circulation (suspension culture), which would help to evaluate the bystander effect induced by the drugs in these cells and its role in causing second malignancies. A549 cells are lung adenocarcinoma cells, which are widely used in *in vitro* models to assess the DNA damage caused by radiation and chemotherapy. Further, BLM and NCS are radiomimetic drugs which induce damage by the formation of free radicals leading to the formation of DNA adducts and are used in the treatment of Hodgkins lymphoma [24], lung cancer and liver cancer [25]. Our results have shown that BLM and NCS induced a bystander response in CCRF-CEM and A549 cells, which was evidenced by the enhanced MN frequency and relative fluorescence intensity of γH2AX foci, measured in the bystander cells (Fig. 1 and 2). Additionally, we observed that the drugs BLM and NCS could not induce a bystander response in promyelocytic leukemic cells (HL-60). This could be attributed to the p53 status, cell survival or due to the high repair capacity, which might confer the characteristics of radio-resistance on them [26]. Furthermore, it was shown that mitochondria play a significant role in both mediating and regulating the bystander effect [27]. The fact that HL-60 cells are devoid of functional mitochondria provides an alternative reason as to why these cells were not able to induce bystander response like lymphoblast and lung adenocarcinoma cells [19].

The enhanced DNA damage induced in the bystander cells could be due to two reasons; first, it may be due to the presence of residual drug in the medium of exposed cells and the second being the bystander response mediated by soluble molecules. While the first was eliminated by HPLC studies, showing below detectable amounts of residual drug in the medium and cell lysate [14], the latter was studied using inhibitors of ROS and NO, as both were shown to be involved in mediating the bystander response. Indeed, ROS are capable of forming DNA damage and DNA single-stranded breaks, which, if not repaired, could be converted to DNA double strand breaks upon collision with the replication fork in S-phase of the cell cycle [28]. NO for example has been proposed to be responsible, at least in part, for bystander effect signal propagation, as it is small, lipophilic, and capable of diffusing freely into cells. Therefore to explore the role of ROS and NO in mediating bystander effects, the free radical scavengers DMSO or C-PTIO were used respectively. Pre-treatment of bystander cells with DMSO or C-PTIO did not alter the base-line MN frequency showing that concentrations of scavengers used are not toxic to the cells used in the study.

Consistent with ROS, involvement of NO in mediating the ionizing radiation induced bystander response [29] has been previously reported. Irradiated conditioned medium transfer experiments in HaCaT cell line, showed that ROS, NO and calcium were all found to be important signaling molecules involved in bystander responses, while ROS and calcium were found to be involved in the production of the bystander signal. However, in A549 cultures, pre-treatment with radical scavengers attenuated the magnitude of bystander response significantly in bystander cells at low concentration and at higher concentration neither DMSO nor C-PTIO altered the magnitude of bystander response. Alternatively, in CCRF-CEM cells, while the pre-treatment of bystander cells with DMSO, reduced the MN frequency significantly in bystander cells, pre-treatment with C-PTIO, did not show any decrease in MN frequency in bystander cells,10 mM of DMSO may not be sufficient to scavenge the ROS generated by high doses of BLM in A549 cells, but it may be effective in CCRF-CEM cells, suggesting that there must be a cell type dependent variation in response to BLM exposure and the resultant release of bystander molecules. Similar results were observed by Shao [30], where DMSO reduced helium 3 induced bystander responses in both skin fibroblast and human glioblastoma cells, but C-PTIO reduced the bystander response only in skin fibroblast cells. In contrast, C-PTIO was found to attenuate the X- irradiated bystander effect, which DMSO failed to do [31]. Whereas in our study, C-PTIO reduced only BLM induced bystander response in A549 and CCRF-CEM cells, it did not in the NCS induced bystander effect in both the cell types. This result was further supported by the COX-2 expression done using flow cytometry, where increased expression of COX-2 was found in BLM exposed cells but not in NCS exposed cells. The observations that (i) NO is known to regulate the expression of IL-8 in some human cells [32] and (ii) NO synthase, which is critical to the biosynthesis of peroxynitrite anions, has been shown to be involved in the regulation of COX-2 expression [33] provide a functional link for the role of NO and COX-2 in mediating the bystander effect. Hence it can be noted that like radiation, chemotherapeutic drugs also induce bystander response and the signaling mechanism for the observed bystander response may be dependent on the cell type. This difference in mechanisms between cells may allow potential exploitation of bystander responses if these are observed in tissue systems. Mechanistic control of the bystander effect could either reduce radiation damage and potential carcinogenesis in normal tissues by reducing bystander signaling or maximize cell sterilization during radiotherapy by amplifying bystander responses in tumors.Taken together, these results expand on current paradigms of inheritance by showing that intercellular interactions can lead to a heritable increase in cellular susceptibility to recombination and suggest that past insults can lead to long-term changes in the risk of de-novo homologous recombination events. If these observations are generally true within normal human tissues, then a single acute exposure to a cancer chemotherapeutic could potentially increase the risk of tumorigenic sequence rearrangements long after the initial exposure.

## Financial Assistance

Department of Science and Technology, Government of India (SR-SO/HS-77/2005).
